# Self-Healing and Self-Adhesive Substrate-Free Tattoo Electrode

**DOI:** 10.3390/ma16093499

**Published:** 2023-05-01

**Authors:** Yuanfen Chen, Xiaoming Yuan, Chunlin Li, Ruicheng Ruan, Hui You

**Affiliations:** 1 School of Mechanical Engineering, Guangxi University, Nanning 530004, China; yuanfenchen@gxu.edu.cn (Y.C.);; 2 Center on Nanoenergy Research, School of Physical Science and Technology, Guangxi University, Nanning 530004, China

**Keywords:** substrate-free tattoos, tattoo electrodes, self-healing, self-adhesive, ECG monitoring

## Abstract

Electronic tattoos have great potential application in the biomedical field; moreover, the substrate-free electronic tattoo offers better comfortability and conformal contact. However, the substrate-free electronic tattoo is more prone to malfunction, including fall off and fracture. In this paper, a self-healing and self-adhesive substate-free tattoo based on PEDOT: PSS is studied and reported. The dry composite electrode will turn into self-healing material while it transforms into hydrogel, and a cut with a width up to 24 μm could be healed in 1 s. In terms of adhesion performance, the substrate-free electrode can hang a 28.2 g weight by a contact area of 8 mm × 8 mm. Additionally, the substate-free electrode could maintain fully conformal contact with porcine skin in 15 days by its self-adhesiveness. When applied as a substrate-free tattoo, the contact impedance and ECG signal measurement performance before and after self-healing are almost the same. At a frequency of 10 Hz, the contact impedance of the undamaged electrode, healed electrode, and Ag/AgCl gel electrode are 32.2 kΩ, 39.2 kΩ, and 62.9 kΩ, respectively. In addition, the ECG signals measured by the undamaged electrode and healed electrode are comparable to that of Ag/AgCl electrode. The self-healing and self-adhesive substrate-free tattoo electrode reported here has broad application in health monitoring.

## 1. Introduction

Electronic skin is an emerging technology for human health monitoring, which is widely applied in wearable electronic devices, human-machine interactions, and other fields [1,2,3]. The electrode is fitted on human skin to collect bioelectrical signals, including electrocardiogram (ECG), electroencephalogram (EEG), electromyography (EMG), and electrooculography (EOG). Continuous ECG monitoring is recommended for cardiovascular patients. However, the commercial Ag/AgCl gel electrode is not suitable for longtime ECG monitoring because the gel would dry and fall off skin and may cause irritation or allergic reaction [4]. Nowadays, researchers are studying dry electrodes which are thin, conductive, air permeable, stretchable, and super-conformal on skin surface, for potential application in long-term ECG monitoring.

Conductive dry electrodes of metals and their derivatives, [5,6,7] and semiconductor materials [8,9,10] are firstly studied. Usually, these rigid materials are patterned on a flexible substrate that could fit on skin. But the mismatch of mechanical material properties would lead to delamination between the conductive layer and substrate. With the rapid development of material science, conducting polymers with high conductivity, flexibility, biocompatibility, and stability have attracted extensive attention from researchers. Lo et al. developed an inkjet-printable (poly (3,4 ethylene dioxythiophene): poly (styrene sulfonate)) (PEDOT: PSS)-based stretchable electrode [11]. The stretchable dry electrodes were printed on a thin polydimethylsiloxane (PDMS) substrate and applied for photoplethysmography (PPG) and electrocardiography (ECG) recording. The substrate offers protection for functional electrodes and adhesiveness to skin. However, substrates would increase the total thickness, and compromise the conformal fit with skin, while substrate-free electronic tattoos offer better comfortability and conformal contact and have received increasing research attention. Kabiri Ameri et al. developed a graphene-based substrate-free electrode and applied it to measure ECG, EEG, and EMG signals [12]. However, the graphene electrodes could only adhere to the skin for a few hours through weak van der Waals force. Recently, Chen et al. reported a PEDOT: PSS-based substrate-free electrode that could improve its conformal contact and interfacial strength with skin through interfacial energy regulation [13]. Interfacial strength was increased by transforming van der Waals force into hydrogen bonds. These reported substrate-free electrodes are thin and could form fully conformal contact with skin, reducing the interfacial contact impedance, thus improving ECG quality. However, without substrate protection, the substrate-free electrode is susceptible to damage and could only rely on weak interfacial interaction to adhere on skin; this increases the possibility of electrode malfunction. Research on self-adhesive and self-healing substate-free electrodes is a promising approach to solve this problem.

PEDOT: PSS, as a conductive polymer, is a promising candidate for self-adhesive and self-healing substate-free tattoo electrodes. The self-adhesive property of the PEDOT: PSS-based electrodes could be improved by adhesive enhancers. In 2020, Zhang [14] et al. obtained a dry electrode with high conductivity and self-adhesiveness for biological signal measurement by adding waterborne polyurethane (WPU) and D-sorbitol to PEDOT: PSS. More recently, Cao et al. reported a dry electrode that could remain self-adhesive to both dry and moist skin. The dry electrode was obtained by adding poly-(vinyl alcohol), tannic acid, and ethylene glycol into PEDOT: PSS [15]. The electrode exhibited 122 S/cm conductivity and 54% mechanical stretch, and had lower skin contact impedance than commercial Ag/AgCl gel electrodes on both dry and sweaty skin. In addition, the self-healing property of the PEDOT: PSS electrode has been widely investigated, either as dry electrodes or hydrogels. In 2017, Zhang [16] et al. reported that a 1 μm thick PEDOT: PSS electrode could convert into self-healing material when moistened with water. In 2019, Kee [17] et al. found that surfactant Triton X-100 improved the self-healing property of PEDOT: PSS. In 2020, Li [18] et al. reported that PEG-400 improved both the self-healing property and flexibility of the PEDOT: PSS electrode. These studies show that PEDOT: PSS could be converted into self-healing material, and this property could be enhanced by additives, though some reported additives are harmful and not suitable for biomedical application. In 2021, Zhou et al. obtained a PEDOT: PSS based self-healing hydrogel and applied it in ECG and EMG measurement [19]. The as-prepared hydrogels exhibited high adhesion on porcine skin, high plastic stretchability, and remarkable self-healing properties. In 2022, Jin et al. reported a 3D printable hydrogel with self-healing and self-adhesive properties [20]. High-quality ECG and EMG signals were obtained by using this hydrogel electrode. These studies laid the foundation for the self-healing PEDOT: PSS electrode, demonstrating that the PEDOT: PSS-based electrode could be self-healing as a hydrogel or trigger self-healing as a dry electrode. However, the previous studies either focused on the self-adhesive property of the dry PEDOT: PSS-based electrode or the self-healing/self-adhesive property of the PEDOT: PSS-based hydrogel. However, the self-adhesive and self-healing substrate-free dry PEDOT: PSS-based electrode has not been reported. The substrate-free dry electrode offers better comfortability and conformal contact with skin, while it is susceptible to damage without substrate protection, and could only rely on its self-adhesiveness to adhere on skin. In order to be applied in long-term health monitoring as a substrate-free dry electrode on skin, the self-healing and self-adhesive properties of the PEDOT: PSS-based substrate-free dry electrode needs more study.

Herein, we reported a self-healing and self-adhesive substrate-free PEDOT: PSS-based tattoo electrode for long-term ECG measurement. The PEDOT: PSS electrode was doped with conductivity and adhesive enhancer glycerin, [21,22] as well as self-healing and adhesive enhancer carboxymethyl cellulose (CMC) [23,24]. The mechanical, electrical, gel transformation, self-healing, and self-adhesive properties of the composite electrode were carefully studied. The damaged substrate-free electrode could be healed immediately once the electrode transformed into hydrogel. In addition, the substrate-free electrode could remain fitted and attached on skin by its self-adhesiveness. The contact impedance and ECG measurement performance of the substate-free electrode before damage and after healing did not change and were comparable to those of the commercial Ag/AgCl gel electrode. The reported self-healing and self-adhesive substate-free electrode could be widely applied in wearable electronic devices, in biomedical and other related fields.

## 2. Materials and Methods

### 2.1. Experimental Materials

PEDOT: PSS (Clevios PH1000) was purchased from Heraeus and used as purchased. Glycerol (AR) was purchased from Kelong Chemical and used as purchased. Carboxymethyl cellulose (CMC) (viscosity: 600–3000 mPa·s) was purchased from Macklin. Phosphate buffer saline (PBS) (PH7.2–7.4) solution was purchased from Macklin.

### 2.2. Electrode Preparation

**Figure A1:**
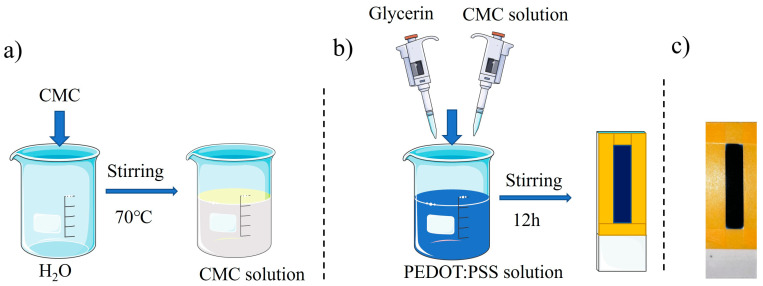
(**a**) Schematic illustration of CMC solution preparation. (**b**) Schematic illustration of PEDOT: PSS electrode preparation. (**c**) Image of the prepared electrode.

The schematic illustration of the PEDOT: PSS electrode preparation process is shown in Figure A1 in Appendix A. We added 0.2 g of CMC to 19.8 mL of deionized water and stirred at 70 °C to obtain a clear 1 wt% CMC solution. The prepared CMC solution and glycerol were then added to the PEDOT: PSS solution to obtain the mixed solution. The content of different solution combinations is shown in Table A1 in Appendix A. The mixed solution was coated on the mold and dried at 60 °C for 12 h in an oven. The dried composite PEDOT: PSS electrode was then peeled off the mold. The electrodes for all the experiments were prepared by adding the same solution volume into the same mold to control the thickness.

### 2.3. Electronic and Mechanical Property Characterization

The thickness of the composite PEDOT: PSS electrodes was measured with a step meter (Bruker Dektak XT, Schramberg, Germany). The electrical conductivity of the electrodes was measured by a four-probe tester (Four Probes Tech, Shenzhen, China). For the resistance changes in different humidity environments, the electrodes were sealed in a closed chamber with controlled humidity, and the resistance was measured by a multimeter. For resistance changes with different PBS buffer additions, 5, 10, 15, and 20 μL PBS buffer were added onto the electrodes with an area of 25 mm × 8 mm, and the resistance was measured by a multimeter. Elastic modulus, elongation at break, and tensile cycle experiments at 20% strain were measured using an electronic universal testing machine (SUNS UTM2502, Dongguan, China).

### 2.4. Healing Property Measurement

To study the healing property of the composite PEDOT: PSS electrodes, the electrodes were cut into two halves using a scalpel, followed by adding DI water or PBS in the cut area, then the healing process was recorded by an image metallographic microscope (53X-V, Shanghai, China). An ultra-depth three-dimensional microscope (Keyence, VHX-6000, Osaka, Japan) was used to observe the conformal contact between the electrode and skin interface before and after healing. The current change during the cut and heal process was measured by an electrochemical workstation (PARSTAT 4000A, Berwyn, IL, USA), and healing efficiency was calculated according to Equation (1).

(1) $$\eta =\frac{I_{healed}-I_{damaged}}{I_{pristine}-I_{damaged}}$$

### 2.5. Adhesion Property Measurement

The adhesion of the electrode was first characterized by the weights it could lift. The composite electrode was attached to a glass slide assembly at one end, and the other end was lifted. A glass slide was added layer by layer until the assembly fell off. The weight of the glass slide assembly that the electrode could lift, and the contact area was measured and recorded. Then, the adhesion of the electrode was characterized by attaching the electrode onto porcine skin; we monitored the contact status continuously for 15 days by using an Ultra-Depth Three-Dimensional Microscope.

### 2.6. Contact Impedance and ECG Signal Measurements

The contact impedances between the human skin and undamaged composite electrode, healed electrode, and Ag/AgCl gel electrode were measured using an electrochemical workstation. The frequency was set in the range of 1–10^4^ Hz with a voltage of 10 mV. The ECG signal was collected by the electrocardiogram monitor (Mindray Heart Beat 3, Shenzhen, China). A pair of Ag/AgCl gel electrodes attached to the left and right legs were applied as the reference electrodes, and an undamaged electrode, healed electrode, and Ag/AgCl gel electrodes attached on the right arm were applied as the working electrodes, respectively. For electrode performance under the wetted condition, the electrode was first attached onto skin, wetted with 100 μL of deionized water or PBS buffer, then the contact impedance and ECG signal were measured. For electrode performance degradation with time, the contact impedance was measured every 6 h for a time period of 12 h; the ECG signal was measured for a time period of 24 h. The collected signal was filtered by a 50 Hz band notch filter to remove power frequency interference, and 20 Hz low-pass filter to remove EMG interference.

## 3. Results and Discussion

### 3.1. Mechanical, Electrical, and Gel Transformation Properties

**Figure 1:**
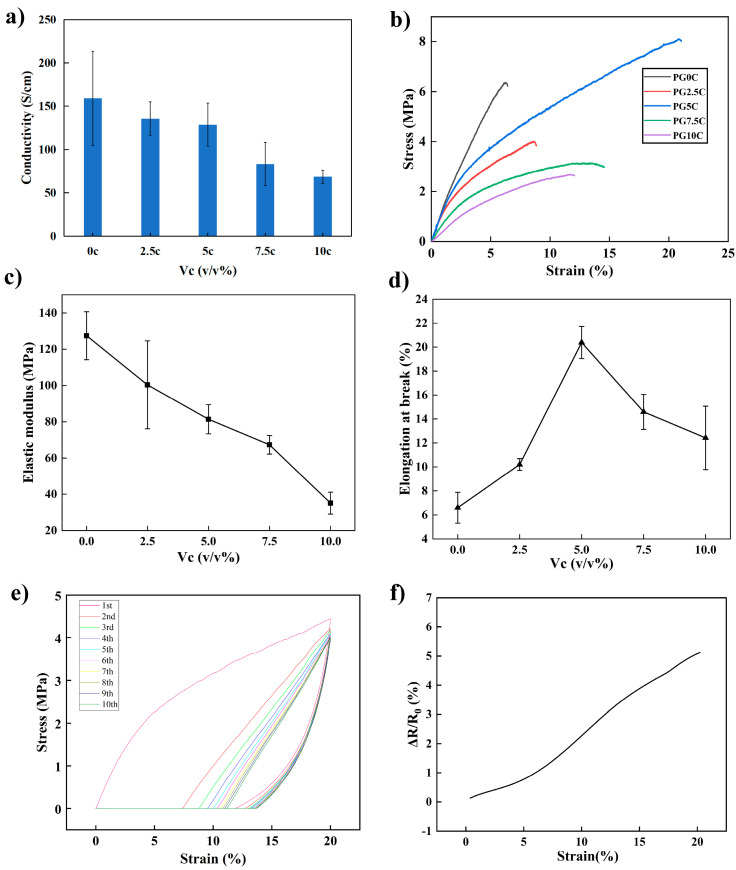
(**a**) Effect of CMC content on composite electrode conductivity. (**b**) Stress-strain curves of the composite electrode with different CMC contents. (**c**) Effect of CMC content on Young’s modulus. (**d**) Effect of CMC content on elongation at break. (**e**) Stress-strain curves of ten tensile relaxation cycles at 20% strain (5 *v*/*v*% CMC solution content). (**f**) The change of resistance as a function of strain (5 *v*/*v*% CMC solution content).

The pure PEDOT: PSS film exhibits low electrical conductivity and mechanical properties. Glycerol was added as the first dopant to improve conductivity. It was added by 2.5 *v*/*v*% according to previous reports [25]. Meanwhile, CMC was added as the second dopant. It could enhance the flexibility and adhesiveness of the composite electrode [24,26]. Composite electrodes with different concentrations of CMC solution were investigated for their mechanical and electrical conductivity. As shown in Figure 1a, the conductivity of PEDOT: PSS with 2.5 *v*/*v*% glycerol was 159 S/cm, and the conductivity decreased as the CMC solution increased. As a non-conductive additive, the increase of CMC solution content dilutes the conductive PEDOT: PSS, thus reducing the conductivity. Figure 1b shows the typical stress-strain curves of the composite PEDOT: PSS electrodes with different CMC solution content, and Figure 1c,d shows the corresponding Young’s modulus and elongation at break, respectively. It could be seen that, as the CMC solution content increased, the Young’s modulus showed a monotonous decreasing trend. That is, the addition of CMC improved the flexibility of the PEDOT: PSS electrode and lowered its stiffness. Meanwhile, as the CMC content increased, the elongation at break increased at first and then decreased, reaching the maximum when the CMC solution content was 5 *v*/*v*%. This may be because that PEDOT: PSS, glycerol, and CMC could be fully cross-linked together at this composition ratio. As an electronic tattoo electrode, it is necessary to have proper mechanical property to avoid deformation and fracture. Taking the electrical conductivity, and mechanical properties into consideration, the composite electrode with 5 *v*/*v*% CMC solution was selected for the subsequent experiments. It had a conductivity of 128.6 S/cm, elongation at break of 21.1%, and elastic modulus of 100 MPa.

To verify the composite electrode’s ability to resist fracture within normal skin stretching, ten tensile cycling experiments at 20% strain were performed. As shown in Figure 1e, the film showed an obvious hysteresis in the first two stretching cycles and cannot return to its initial state; however, it stabilized at the subsequent cycles. In order to study conductivity stability of the electrode during the stretching process, the resistance change as a function of strain was studied. As shown in Figure 1f, the change of resistance was about 5% within 20% strain. These results showed that the optimized film could be subjected to successive multiple tensile cycling without damage, and the conductivity could keep stable within a normal skin stretch.

**Figure 2:**
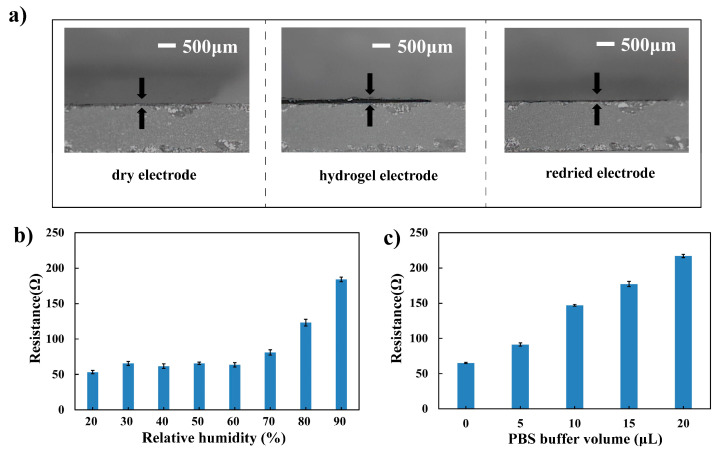
(**a**) Image of the composite electrode at dry, hydrogel, and redried state; (**b**) resistance changes with humidity; (**c**) resistance changes with different PBS buffer addition.

**Figure A2:**
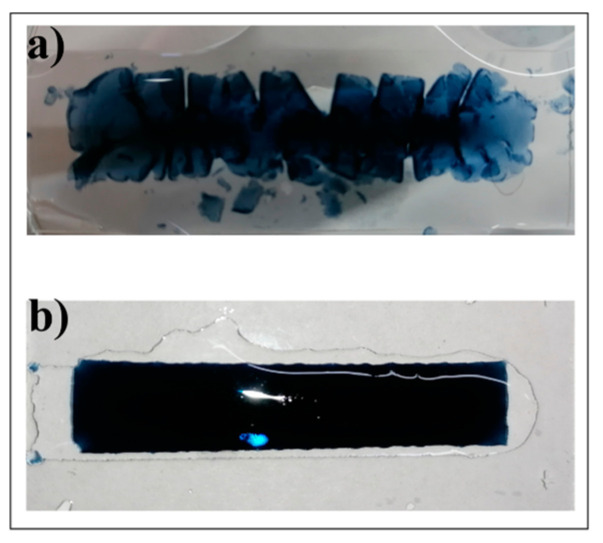
(**a**) Pure PEDOT: PSS film in water. (**b**) Composite PEDOT: PSS film in water.

In addition, the pure PEDOT: PSS film would disperse rapidly in water, but the composite electrode could transform into a stable hydrogel in water, as shown in Figure A2 in Appendix A. The hydrogel electrode could turn back into a dry electrode after the water has evaporated. Figure 2a showed the dry electrode, hydrogel electrode, and redried electrode. The swelling ratio in thickness direction was about 2. The dry electrode and hydrogel transformation property agreed with the previously reported pure PEDOT: PSS electrode and PEDOT: PSS/PVA composite electrode. The pure PEDOT: PSS electrode had a swelling ratio up to 7 [27], and the PEDOT: PSS/PVA composite electrode had a swelling ratio of 5.85 [13]. These results showed that the swelling ratio of the PEDOT: PSS-based electrode would be affected by the enhancers.

When applied on skin as a substrate-free electrode, the electrode would undergo different humidity and sweat environments. The resistance changes of the electrode under different humidity environments were first studied. As shown in Figure 2b, the resistance of the composite electrode would keep stable when humidity was lower than 60 RH% and would increase with higher humidity. As humidity increased from 60 RH% to 90 RH%, the conductivity would increase from 63.57 ± 2.9 to 184.1 ± 3.3 Ω. The resistance changes with different PBS buffer addition were also investigated to evaluate the electrode conductivity stability under different sweat environments. As shown in Figure 2c, the resistance of the composite electrode would decrease as the PBS buffer volume added increased. With 20 μL PBS buffer added, the resistance of the electrode would increase from 65.1 ± 0.9 to 216.8 ± 2.2 Ω. It could be seen that the resistance of the composite electrode would be affected by the humidity and PBS buffer addition. As the impedance in ECG signal measurement was affected by the skin impedance, skin-electrode interfacial impedance, and electrode impedance, even though the skin impedance and skin-electrode interfacial impedance were much higher than the electrode impedance, the influence of the humidity and PBS addition on the total impedance and ECG signal quality will be studied in the following sections.

### 3.2. Healing Properties

**Figure 3:**
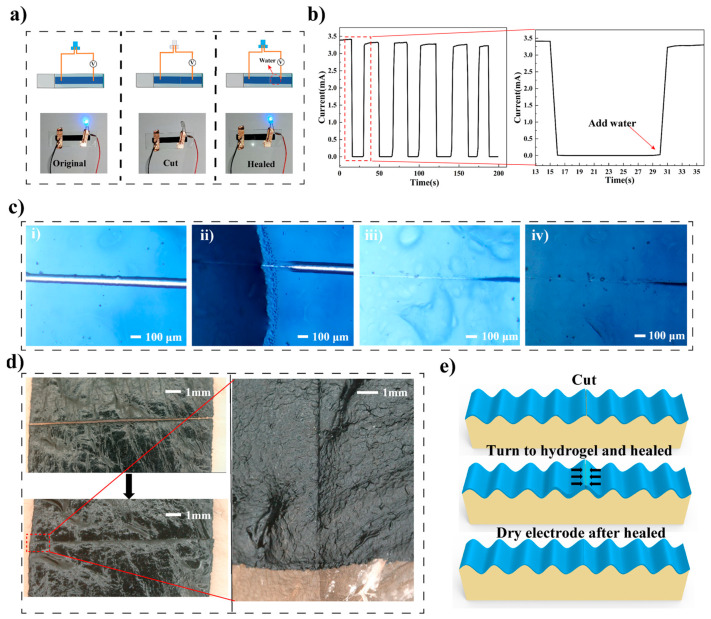
(**a**) LED lit up, turned off, and lit up again during the cut and heal process. (**b**) Current as a function of time during multiple cuts and heals, the enlarged figure shows the first cut and heal cycle. (**c**) Images of the healing process: (**i**) the electrode was cut through; (**ii**) the electrode healed after being wet (left dark side was wet by water and healed); (**iii**) healed hydrogel; (**iv**) healed dry electrode. (**d**) Left: composite electrode on porcine skin before and after healing; Right: conformal contact after healing. (**e**) A schematic representation of the healing process.

The healing property of the PEDOT: PSS composite electrode was first characterized based on electrical property recovery. As shown in Figure 3a, the LED lit up after the power switched on, and turned off when the electrode was cut through using a scalpel; the electrode thickness was 15.6 ± 0.7 μm, and the through cut width was about 24 μm. Then, the LED lit up again after 10 μL deionized water was added to the cut area, indicating that after wetting by water, the electrical property of the cut electrode was recovered. Figure 3b shows the current as a function of time during multiple cuts and wetting. It could be seen that the current dropped immediately after the cut and recovered immediately after water was added. As shown in the enlarged figure in Figure 3b, the current recovery time was about 1 s, and we defined this time as the electrical healing time of the composite electrode. According to Equation (1) shown in the Experimental Section, the conductivity healing efficiency was calculated to be 94% after five repeated cuts. The healing performance was comparable with the previous reported PEDOT: PSS-based electrodes. With Triton X-100 as a healing agent, which was toxic and not suitable for health monitoring, the healing time of the PEDOT: PSS composite electrode was around 1 s, and the retaining power output after cut-and-heal was 85% [17]; while the PEDOT: PSS/PEG-400 electrode showed healing times ranging between 50 and 800 ms, and healing efficiency was reported to be close to 100% [18].

**Figure A3:**
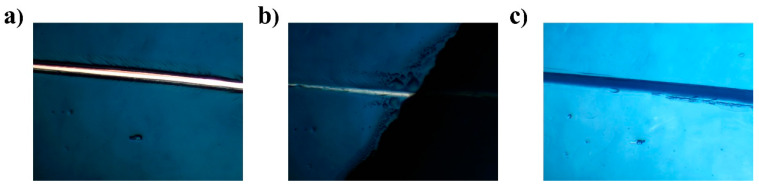
Images of the healing process: (**a**) the electrode was cut through; (**b**) the electrode healed after being wet (left dark side was wet by PBS and healed); (**c**) healed dry electrode.

**Figure A4:**
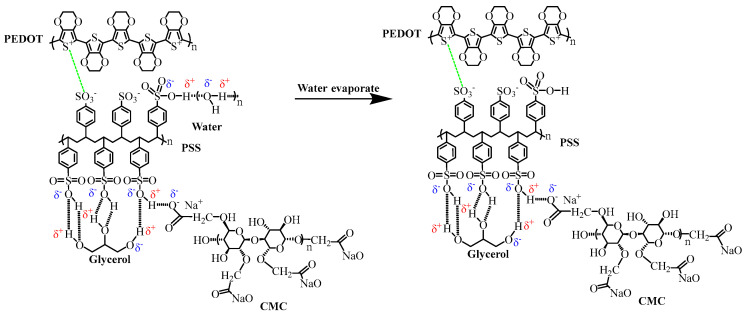
The possible hydrogen bond interactioan between PEDOT:PSS, CMC, and Glycerol.

The healing process of the composite electrode was then recorded and shown in Figure 3c. A crack could be clearly observed in the middle of the electrode after being cut through (Figure 3c(i)). The cut area healed within 1 s after deionized (DI) water was added and the electrode turned into hydrogel (Figure 3c(ii)). Then, the healed hydrogel (Figure 3c(iii)) turned into healed dry electrode (Figure 3c(iv) as the water evaporated. The healing of the composite electrode triggered by phosphate buffer saline (PBS) is shown in Figure A3 in Appendix A. The healing process of the composite electrode triggered by DI water and PBS was similar, which indicated that the composite electrode could be triggered to heal by human sweat on skin. Meanwhile, as shown in Figure 3d, the substrate-free electrodes on porcine skin could be healed after being cut through and keep conformal contact even after healed. Considering that the composite electrode could transform between a dry electrode and hydrogel, the healing process was schemed and is shown in Figure 3e. After water was added to the cut area, the dry electrode swells and turns into hydrogel, and the swelling brings the two cut halves closer to each other. In addition, the large amount of hydrogen bonds between hydroxyl groups in water, CMC, and glycerol, as well as the hydrogen bonds between the hydroxyl groups and sulfonic acid groups in PSS chains, bring the PSS chains closer and even cross-link with each other, healing the hydrogel. The possible hydrogen bond interaction is shown in Figure A4 in Appendix A. As water evaporates, the healed hydrogel turns into healed dry electrode.

### 3.3. Adhesion Properties

**Figure 4:**
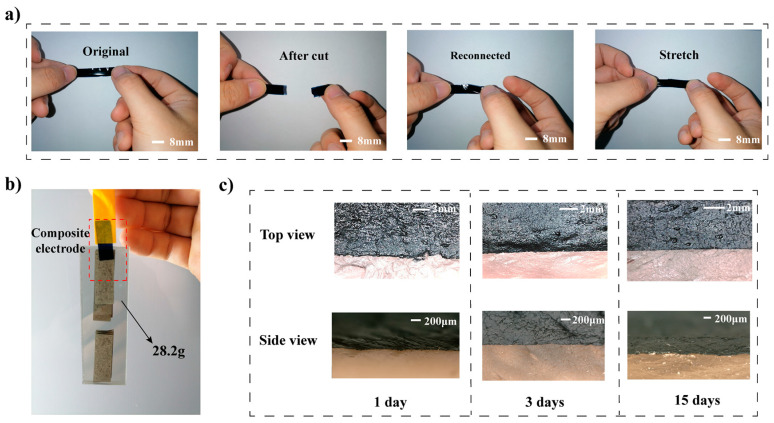
(**a**) Images of the composite electrode at its original state, cut into halves, reconnected, and stretched. (**b**) Composite electrode hanging a glass assembly. (**c**) Electrode fitted on porcine skin from top view and side view, on the 1st, 3rd, and 15th day.

The self-adhesion property of the substrate-free composite electrode was studied. As shown in Figure 4a, the electrode could be reconnected after being cut apart, and relying on the electrode’s self-adhesiveness, the reconnected electrode could be stretched without being disconnected. As shown in Figure 4b, the substrate-free electrode was attached to a glass slide assembly at one end, and the adhesive tape at the other end. The electrode can hang the 28.2 g glass assembly through its adhesiveness by a contact area of 8 mm × 8 mm, corresponding to 44.1 g/cm^2^, comparable to the previously reported PEDOT: PSS-based dry electrodes reported by Zhang et al., which could bear an object of 250 g with 2.5 × 2.5 cm^2^ (Corresponding to 40 g/cm^2^) [14]. Figure 4c showed the electrode–porcine skin interface for 15 days. As time progresses, the porcine skin shrunk and deformed due to dehydration, but the electrode adapted to the change and maintained conformal contact with the porcine skin even at the edge of the interface. The result showed that the substrate-free electrodes could stay well-attached and fitted on skin for days, showing great potential for long-term health monitoring.

### 3.4. Contact Impedance and ECG Signal Measurement

**Figure 5:**
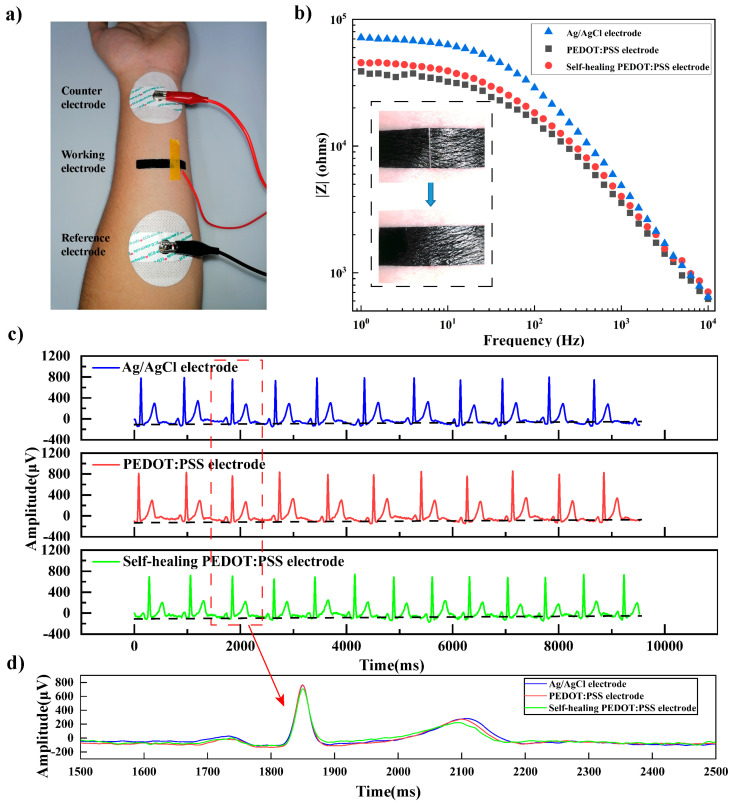
(**a**) Contact impedance measurement setup. (**b**) Contact impedance of the undamaged electrode, healed electrode, and Ag/AgCl gel electrode, inset was a photograph of the electrode before and after healing on human skin. (**c**) ECG signals collected by the three different electrodes. (**d**) The electrocardiographic signal taken one cycle from Figure 5c.

The composite electrode was then applied as a substrate-free tattoo electrode on human skin for health monitoring. The substrate-free tattoo electrode could stay fit on human skin by its self-adhesiveness. Figure 5a shows the contact impedance measurement setup. The undamaged electrode, healed electrode, and Ag/AgCl gel electrode were used as the working electrode, respectively. Additionally, two Ag/AgCl gel electrodes were used as the reference electrode and counter electrode. The contact impedances are shown in Figure 5b. The inset figure showed the electrode on human skin before and after healing. It could be seen that the contact impedance of Ag/AgCl at 1–10^3^ Hz was significantly higher than those of the undamaged and healed electrode. The contact impedance of Ag/AgCl, undamaged electrode, and healed electrode at 10 Hz were 62.9 kΩ, 32.2 kΩ, and 39.2 kΩ, respectively.

The ECG signal measured by the undamaged electrode, healed electrode, and Ag/AgCl electrode is shown in Figure 5c,d. It could be seen that the P wave, QRS band and T wave could be clearly distinguished for signals measured by all three electrodes. The amplitudes of the Ag/AgCl gel electrode and undamaged electrode almost overlapped with each other, and the amplitudes of the healed electrode was slightly lower, but still comparable to that of the undamaged electrode. The results showed that the healed electrode could maintain contact impedance and ECG measurement performance almost the same as the undamaged ones, demonstrating the potential of the reported PEDOT: PSS-based electrode as a substrate-free, self-healing and self-adhesive tattoo electrode for ECG measurement.

**Figure 6:**
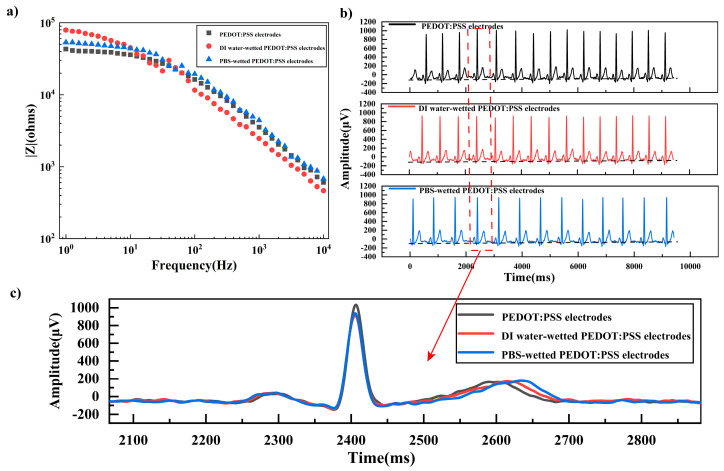
(**a**) Electrode-skin contact impedance of the dry electrode and wetted electrodes. (**b**) ECG signals collected by the dry electrode and wetted electrodes. (**c**) One ECG signal taken from Figure 6b.

To evaluate the effect of water or sweat on the performance of the composite electrodes, the contact impedance and ECG signals of the electrodes wetted with deionized water and PBS buffer were tested. As shown in Figure 6a, the contact impedance of the electrode at frequencies lower than 200 Hz increased after wetting with both deionized water and PBS buffer, while the contact impedance at frequencies higher than 300 Hz decreased after wetting. This might be because the presence of water or PBS buffer increased the electrode impedance, as shown in Section 3.1, corresponding to the contact impedance at lower frequency, while the water or PBS buffer would increase the interfacial contact between the electrode and skin, reducing the interfacial impedance, and corresponding to the contact impedance at higher frequency. The contact impedances for wetting with deionized water and PBS buffer were almost overlapping with each other, showing that ions in PBS buffer had little effect on the contact impedance. Figure 6b,c shows the ECG signals measured by the dry electrode and wetted electrodes. The signals of the wetted electrodes were comparable to that of the dry electrode, indicating that the PEDOT: PSS electrode had good ECG acquisition capabilities in both dry and wetted conditions.

**Figure A5:**
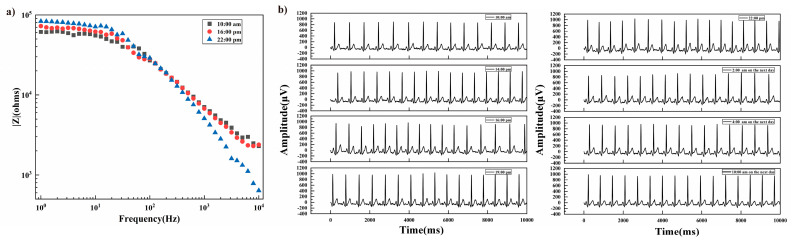
(**a**) Impedance test over 12 h. (**b**) ECG signal measurement over 24 h.

To evaluate the contact impedance stability and the ECG signal measurement performance degradation with time, the contact impedance and ECG signal were measured over hours. As shown in Figure A5a in Appendix A, the impedance of the PEDOT: PSS electrodes fluctuated slightly over 12 h. At a frequency lower than 100 Hz, the contact impedance of the electrode increased slightly with time, while at a frequency higher than 100 Hz, the contact impedance decreased with time. As shown in Figure A5b, the ECG signals measured over 24 h were comparable. The P wave, QRS band and T wave could be clearly distinguished for all the signals. These results showed that the substrate-free, self-adhesive, and sealing-healing PEDOT: PSS electrode has potential for health monitoring in a time period spanning hours.

To better evaluate the contribution of this work, related work was compared in terms of electrode type, adhesion property, self-healing property, and bioelectrical signals measurement performance. As shown in Table 1, while self-adhesive and self-healing properties were widely studied for hydrogel electrodes, the previous work on substrate-free dry electrode focused either on the self-adhesive property or the self-healing property. The current work reported a substrate-free dry electrode that is both self-adhesive and self-healing, with the self-adhesive strength comparable to the previously reported substrate-free dry electrodes, and with the self-healing property comparable to the hydrogel electrodes.

## 4. Conclusions

In this paper, a substrate-free, self-healing, and self-adhesive tattoo electrode was studied and reported. The mechanical and electrical performance of the composite electrode was first studied to optimize the electrode composition. The optimized electrode had a conductivity of 128.6 S/cm, elongation at break of 21.1%, elastic modulus of 100 MPa, and stable gel transformation property. The self-healing studies showed that a cut through the electrode, with gaps as wide as 24μm, could be healed once it transformed into hydrogel, and the electrical property could be recovered. The substrate-free electrode could also be healed on porcine skin and human skin. For self-adhesive performance, the substrate-free electrode could hang a weight of 28.2 g through its adhesiveness by a contact area of 8 mm×8 mm. Continuous monitoring of the PEDOT: PSS electrode attached to porcine skin showed that the electrode could adapt to skin changes and maintain conformal contact for 15 days. The substrate-free PEDOT: PSS electrode had contact impedance of about 32.2 kΩ, which was about 1/2 of that of the Ag/AgCl electrode. In terms of ECG measurement, the quality of the ECG signal collected by both the undamaged and healed electrodes was comparable with that of the commercial Ag/AgCl gel electrode. In addition, the ECG measurement performance of the reported electrode was stable in wetted environment, and there was no obvious performance degradation within 24 h. The substrate-free, self-healing, and self-adhesive electrode reported in this paper shows great potential in physiological signal monitoring in wearable electronics.

## Figures and Tables

**Table 1:** Comparison table of crucial properties for related works.

Electrode Types	Adhesion Properties	Healing Properties	Bioelectrical Signals Measurement	Research Work
Hydrogel electrodes	1.96 ± 0.11 N/cm^2^	Healing time: 2 s Healing efficiency: 100%	ECG, EMG	Ref. [19]
Hydrogel electrodes	13.0 ± 0.8 kPa	Could be self-healed	Could be direct printed on tissues	Ref. [20]
Flexible dry electrodes	-	-	PPG, ECG	Ref. [11]
Substrate-free dry electrode	Van der Waals force: 18 mJ·m^−2^	-	ECG, EEG, EMG	Ref. [12]
Substrate-free dry electrode	Orthogonal direction: 52.2 g/cm^2^ Tangential direction: 11.8 g/cm^2^ 90 °C peeling direction: 1.6 ± 0.2 N/m	-	ECG	Ref. [13]
Substrate-free dry electrode	Tangential direction: 40 g/cm^2^	-	ECG, EEG, EMG	Ref. [14]
Substrate-free dry electrode	Wet skin: 0.28 N cm^−1^; Dry skin: 0.32 N cm^−1^	-	ECG, EMG	Ref. [15]
Substrate-free dry electrode	-	Healing time: 150 ms Healing efficiency: 100%	-	Ref. [16]
Substrate-free dry electrode	-	PEDOT: PSS/PEG-400 healing times: 50–800 ms; Healing efficiency: close to 100%	-	Ref. [18]
Substrate-free dry electrode	Tangential direction: 44.1 g/cm^2^	Healing time: 1 s Healing efficiency: 94%	ECG	This work

## Data Availability

Data is contained within the article or Appendix A.

## Appendix A

**Table A1:** The content of different solution combination.

Code	PEDOT: PSS Content (*v*/*v*%)/Solvent Volume	Glycerol Content (*v*/*v*%)/Solvent Volume	CMC Solution Content (*v*/*v*%)/Solvent Volume
PG0C	97.5 (3000.00 μL)	2.5 (76.92 μL)	0 (0 μL)
PG2.5C	95.0 (3000.00 μL)	2.5 (78.94 μL)	2.5 (78.94 μL)
PG5.0C	92.5 (3000.00 μL)	2.5 (81.08 μL)	5.0 (162.16 μL)
PG7.5C	90.0 (3000.00 μL)	2.5 (83.33 μL)	7.5 (250 μL)
PG10.0C	87.5 (3000.00 μL)	2.5 (85.71 μL)	10.0 (342.85 μL)
